# Effects of grazing intensity on plant community fungal diseases in an alpine grassland

**DOI:** 10.3389/fpls.2026.1900475

**Published:** 2026-08-03

**Authors:** Zhen Tian, Xiaoxia Yang, Quanmin Dong, Tao Chen

**Affiliations:** 1 State Key Laboratory of Herbage Improvement and Grassland Agro-Ecosystems; Center for Grassland Microbiome; College of Pastoral Agricultural Science and Technology, Lanzhou University, Lanzhou, China; 2 State Key Laboratory of Plateau Ecology and Agriculture, Qinghai Academy of Animal and Veterinary Science, Qinghai University, Xining, China

**Keywords:** alpine meadow, foliar diseases, grazing, herbivores, nutrition, plant-pathogen interactions, soil fungal pathogens

## Abstract

Fungal pathogens are a vital component of microbial communities and can substantially reduce plant productivity. In grasslands, pathogens and their host plants frequently coexist with large grazing herbivores. However, it remains unclear whether and how grazing alters pathogen transmission and prevalence, particularly when integrating soil and foliar pathogens. Here, we conducted an 8-year field experiment in alpine grasslands on the Qinghai-Tibetan Plateau to examine how soil pathogens and foliar diseases respond to grazing intensity, and to assess the relationship between them. Our results showed that light grazing significantly increased the incidence and severity of plant community foliar diseases, while moderate and heavy grazing significantly reduced them. Similarly, light grazing significantly increased the relative abundance of soil pathogens. Notably, we found a strong positive relationship between soil pathogen abundance and foliar disease occurrence. Mechanistically, light grazing indirectly exacerbated foliar diseases through increases in soil pathogen abundance, whereas moderate and heavy grazing directly suppressed them. Overall, our findings highlight the distinct effects of different grazing intensities on soil pathogens and foliar diseases, and further provide experimental evidence that soil pathogens may play an important role in foliar disease dynamics in grassland ecosystems.

## 1 Introduction

Fungal pathogens are ubiquitous across both aboveground and belowground ecosystems (Fisher et al., 2012; Delgado-Baquerizo et al., 2020). They can impair plant photosynthesis and nutrient acquisition by infecting plant tissues, thereby substantially reducing plant biomass (Allan et al., 2010; Parker et al., 2015; Wang and Li, 2025). For instance, well-known fungal pathogenic genera such as *Alternaria* and *Fusarium* have been shown to pose substantial threats to agricultural production and soil health worldwide (Parry et al., 1995; Peng et al., 2023). However, fungal pathogen abundance and diversity are highly sensitive to environmental change (Singh et al., 2023). Recent studies have shown that warming, nitrogen (N) inputs, and land management intensification can increase the relative abundance and distribution of soil fungal pathogens (hereafter “soil pathogens”), and may increase the risk of foliar fungal disease (hereafter “foliar diseases”) outbreaks under future scenarios (Delgado-Baquerizo et al., 2020; Li et al., 2023; Case et al., 2025). Therefore, understanding soil and foliar pathogen dynamics under climate change and human activities is crucial for maintaining ecosystem structure and functioning.

Grasslands cover approximately 40% of the Earth’s terrestrial surface and provide essential ecosystem services (Bai and Cotrufo, 2022; Pillar and Winck, 2026). Livestock grazing is one of the primary human activities in grasslands and plays a key role in shaping plant community structure and soil properties (Pichon et al., 2025). In recent decades, growing demand for livestock products has intensified grazing pressure worldwide (Herrero‐Jáuregui and Oesterheld, 2018). In grazed systems, livestock, host plants, and pathogens commonly coexist, such that increasing grazing intensity may profoundly influence both soil pathogens and foliar diseases (Liu et al., 2022). There are several potential mechanisms that can explain how grazing affects foliar diseases. At the host species level, grazers reduce diseases by removing infected tissues and pathogen propagules, thereby limiting transmission, whereas grazing-induced tissue damage facilitates pathogen entry and increases infection risk (Daleo et al., 2009; Liu et al., 2022). In addition, grazing can reduce foliar diseases by inducing plant defense responses (Stout et al., 2006). At the community level, grazing can regulate foliar disease transmission and prevalence by altering the abundance of susceptible host species (Borer et al., 2009; Liu et al., 2022). However, current research has largely focused on aboveground foliar diseases (Liu et al., 2021, 2022). Soil serves as a major reservoir of plant pathogens and harbors numerous potentially aggressive and invasive taxa (Du et al., 2022), yet soil pathogens in grasslands remain poorly understood. In particular, whether changes in soil pathogens influence foliar diseases remains largely unknown.

Available soil nutrients are key determinants of both soil pathogens and foliar diseases (Veresoglou et al., 2013). Nutrient enrichment can promote pathogen growth, reproduction, and the infection process (Huber and Watson, 1974). Recent studies have shown that N addition can increase the relative abundance of soil pathogens in N-limited grasslands (Lekberg et al., 2021). In addition, high N conditions may increase host susceptibility by altering plant growth-defense trade-offs and suppressing immune responses (Mitchell et al., 2003; Cappelli et al., 2020). Grazing is one source of soil N in grasslands through excreta return, although its effects vary with grazing intensity (Bardgett and Wardle, 2003; Li et al., 2025). For example, appropriate grazing may enhance nutrient turnover by stimulating plant compensatory growth and rhizosphere activity, whereas heavy grazing may reduce nutrient accumulation through decreased litter resource inputs and soil compaction (Zhou et al., 2017; Duan et al., 2023). Therefore, different grazing intensities may exert distinct impacts on soil pathogens and foliar diseases through differential effects on soil nutrients, yet experimental evidence remains lacking.

Here, we conducted a long-term grazing experiment in an alpine grassland on the Qinghai-Tibetan Plateau to investigate whether and how varying grazing intensities affect soil pathogens and foliar diseases. In our study, we quantified foliar disease incidence and severity at both the population and community levels. We then identified and evaluated soil pathogens using high-throughput sequencing, real-time quantitative polymerase chain reaction (qPCR), and the FUNGuild database. In addition, we measured soil nutrient concentrations. Specifically, our experiment investigated: (a) the impact of grazing intensity on foliar diseases and soil pathogens, (b) the relationship between soil pathogens and foliar diseases, and (c) the potential mechanisms through which different grazing intensities affect soil pathogens and foliar diseases.

## 2 Materials and methods

### 2.1 Study site

Our experiment was conducted in an alpine grassland in Haiyan County, Qinghai Province, China (36°56′N, 100°53′E; 3,200 m above sea level). The mean annual temperature is 1.4 °C and annual precipitation is approximately 400 mm, with rainfall largely concentrated between May and September, reflecting a typical plateau climate (Liu et al., 2023). The soil is classified as clay loam, and the plant community is dominated by *Kobresia humilis* and *Carex aridula* (Yang et al., 2019). The dominant grazing livestock in the study area are yaks and sheep.

### 2.2 Grazing experiment

In 2014, a grazing experiment was established in a flat alpine meadow, and the site was fenced to exclude large wild herbivores. The experiment followed a randomized complete block design with three blocks. Each block contained four treatments: a control without grazing (CK) and three grazing intensity levels (light, LG; moderate, MG; and heavy grazing, HG). Each treatment consisted of three replicate plots, resulting in a total of 12 plots. The details of sheep density, forage utilization rate, and plot area for each grazing intensity are shown in **Table 1**. One sheep unit (SU) is generally defined based on an adult sheep with a live weight of 50 ± 2 kg. However, in our study, the sheep used were one-year-old individuals with an average live weight of approximately 25 ± 2 kg. Therefore, two sheep were considered equivalent to one SU based on this weight difference. Stocking rate (SU ha⁻¹) was calculated as the number of sheep units divided by the enclosure area. Grazing was conducted annually during the growing season (June-October), with each monthly grazing event lasting approximately 10 days. No supplementary feed was provided during the grazing period, but drinking water was supplied daily (Yang et al., 2019). In addition, livestock grazing was the only treatment applied in the experimental plots, with no other management practices (e.g., mowing or fertilization). Prior to soil sampling and plant foliar disease assessments, the grazing treatments had been applied continuously for eight years, allowing sufficient time for treatment effects to manifest.

**Table 1:** Details of the grazing intensity experimental design.

Treatment	Number of sheep	Stocking rate unit (SU ha⁻¹)	Defoliation level (%)	Area of plot (hm^2^)
CK	0	0	0	0.05
LG	3	2.27	30-35	0.66
MG	4	3.92	50-55	0.51
HG	5	6.41	65-70	0.39

CK, fenced ungrazed control that completely excluded livestock grazing, trampling, and dung input; LG, Light grazing; MG, Moderate grazing; HG, Heavy grazing.

### 2.3 Sampling

In August 2022 (peak growing season), three 0.5 m × 0.5 m quadrats were randomly established in each plot. We recorded all host plant species within each quadrat and assessed foliar disease types (e.g., leaf spot, rust, and powdery mildew) and severity. Briefly, for each host plant species in each quadrat, five individuals were selected from the four corners and the center of the quadrat, and five leaves per individual were assessed (25 leaves in total). Specifically, whenever possible, two leaves were selected from the lower part, two from the middle part, and one from the upper part of each individual; when fewer than 25 leaves were available, all leaves were assessed. Disease severity index was assessed following Liu et al. (2022) as the percentage of leaf area covered by fungal lesions. Subsequently, we collected 15 infected leaves per host plant species and identified the pathogen using an upright light microscope (Primo Star, Carl Zeiss, Germany). Finally, three soil cores (0–10 cm depth, 5 cm diameter) were collected within each quadrat and homogenized into a composite sample after passing through a 2-mm sieve to remove stones and plant residues. The composite sample was then divided into two subsamples: one was stored at -80 °C for soil pathogen community analysis, and the other was air-dried for soil property analyses. Soil total nitrogen (TN), ammonium nitrogen (NH_4_^+^-N), nitrate nitrogen (NO_3_^--^N), total phosphorus (TP) and available phosphorus (AP) were measured using a discrete auto analyzer (SmartChem 450, AMS, Italy). Soil organic carbon (SOC) was determined by the potassium dichromate oxidation method.

### 2.4 Plant community foliar disease measurements

We used the community mean disease incidence and severity to assess the occurrence of foliar fungal diseases at the plant community level:


$$\text{Community foliar disease incidence}\left(\%\right)=\frac{N_{\text{infected}}}{N_{\text{infected}}+N_{\text{healthy}}}\times 100\%$$

$$\text{Community foliar disease severity}=\frac{\sum_{\text{i}=1}^{S}DI_{i}}{S}$$

where *N*_infected_ is the number of infected leaves, *N*_healthy_ is the number of healthy leaves, *DI_i_* denotes the disease severity of the *i*-th plant species, and *S* is the total number of host plant species within the quadrat.

### 2.5 Soil pathogen measurements

We used amplicon sequencing and the FUNGuild database to characterize the composition and relative abundance of the potential soil fungal pathogen community. Genomic DNA was isolated from 0.25 g of each soil sample using DNeasy PowerSoil Kit (Qiagen, Germany) following the manufacturer’s instructions, and the fungal ITS2 region was amplified using the forward primer ITS3 (5’-GCATCGATGAAGAACGCAGC-3’) and the reverse primer ITS4 (5’-TCCTCCGCTTATTGATATGC-3’) (White et al., 1990). Amplicons were sequenced on an Illumina MiSeq platform (Illumina Inc., USA), and raw reads were processed using the DADA2 pipeline in R (Callahan et al., 2016) to generate amplicon sequence variants (ASVs). Further details are provided in **Supplementary Methods S1**. We then annotated ASVs using the FUNGuild database (Nguyen et al., 2016) to identify putative soil fungal plant pathogens. Only taxa assigned as obligate pathogens or taxa with multiple trophic modes and classified with ‘probable’ or ‘highly probable’ confidence were retained (Nguyen et al., 2016). Pathogen richness was quantified as the number of pathogen-associated ASVs, and relative abundance was calculated as the proportion of pathogen-associated reads relative to the total fungal sequences.

In addition, we used qPCR to quantify the absolute abundance of *Alternaria* in soil. *Alternaria* was selected because it was identified as the foliar pathogen of the two dominant species with the highest disease incidence and severity in our study plots (**Supplementary Table 1**, **Supplementary Figure 1**). The absolute abundance of *Alternaria* was estimated using primers Dir1ITSAltF (5′-TGTCTTTTGCGTACTTCTTGTTTCCT-3′) and Inv1ITSAltR (5′-CGACTTGTGCTGCGCTC-3′), which are widely applied for the specific quantification of *Alternaria* spp. (Delgado-Baquerizo et al., 2020), on an ABI 7300 Real-Time PCR System (Applied Biosystems, USA) with SYBR Green chemistry. Detailed information on the qPCR procedures is provided in **Supplementary Methods S2**.

### 2.6 Statistical analyses

All analyses were performed in R version 4.4.0 (R Core Team, 2024). Prior to analysis, all variables were standardized to *Z*-scores to place them on comparable scales. To assess the impact of grazing intensity on community foliar disease incidence and severity, we constructed linear mixed-effects models (LMMs) using the *lmer* function in the ‘*lme4*’ package. In these models, grazing intensity (LG, MG, HG) was included as a fixed effect, and plot was included as a random effect. We then conducted *post hoc* multiple comparisons using Tukey’s HSD test in the ‘*agricolae*’ package whenever fixed effects were significant. The same approach was used to examine the effects of grazing intensity on soil pathogens and soil properties. Soil pathogen response variables included the total relative abundance and richness of all soil pathogens, the relative abundance of the three dominant genera (*Fusarium*, *Paraphoma*, and *Alternaria*) observed in our study (**Supplementary Figure 2**), and the absolute abundance of *Alternaria*. Furthermore, we used linear models to examine the relationships between community foliar disease incidence and severity and various soil pathogen variables.

To explore the potential mechanisms by which different grazing intensities affect community foliar disease, we constructed three piecewise structural equation models (SEMs) using the ‘*piecewiseSEM*’ package. Given the strong correlation between community foliar disease incidence and severity, we applied principal component analysis (PCA) to derive a composite index, referred to as ‘community foliar disease’. The first principal component (PC1) explained 93.4% of the variance and was used as the response variable in subsequent SEMs. To simplify model structure and reduce multicollinearity, variables with high collinearity and non-significant effects were sequentially removed until a satisfactory model fit was achieved. Model fit was evaluated using Fisher’s C statistic and the associated *p*-value, with a *p* > 0.05 indicating an acceptable fit.

## 3 Results

### 3.1 Population-level foliar diseases and soil pathogens

A total of 33 plant species were recorded in our study plots, and 21 were identified as hosts for leaf spot diseases (**Supplementary Table 1**). The two dominant species, *C. aridula* and *K. humilis*, were the most common hosts, exhibiting the highest disease levels (**Supplementary Table 1**, **Supplementary Figure 1**); both exceeded 70% in incidence and 30 in severity in all grazing treatments. Interestingly, leaf spot diseases of *C. aridula* and *K. humilis* were both caused by *Alternaria alternata* (**Supplementary Figure 1**). In addition, foliar diseases in grasses were mainly caused by *Phoma* sp., *Septoria* sp., and *Parastagonospora nodorum*, whereas foliar fungal diseases of forbs were caused by a wider diversity of pathogen species (**Supplementary Table 1**).

We assigned 1,565 ASVs to fungal functional guilds using the FUNGuild database, of which 291 ASVs were identified as potential soil pathogens, accounting for 18.59% of the total fungal ASVs. On average, *Fusarium*, *Paraphoma*, and *Alternaria* were the dominant genera within the potential soil pathogen community, with similar relative abundances (0.6%, 0.6%, and 0.5%, respectively) (**Supplementary Figure 2**).

### 3.2 Community-level foliar diseases and soil pathogens

**Figure 1:**
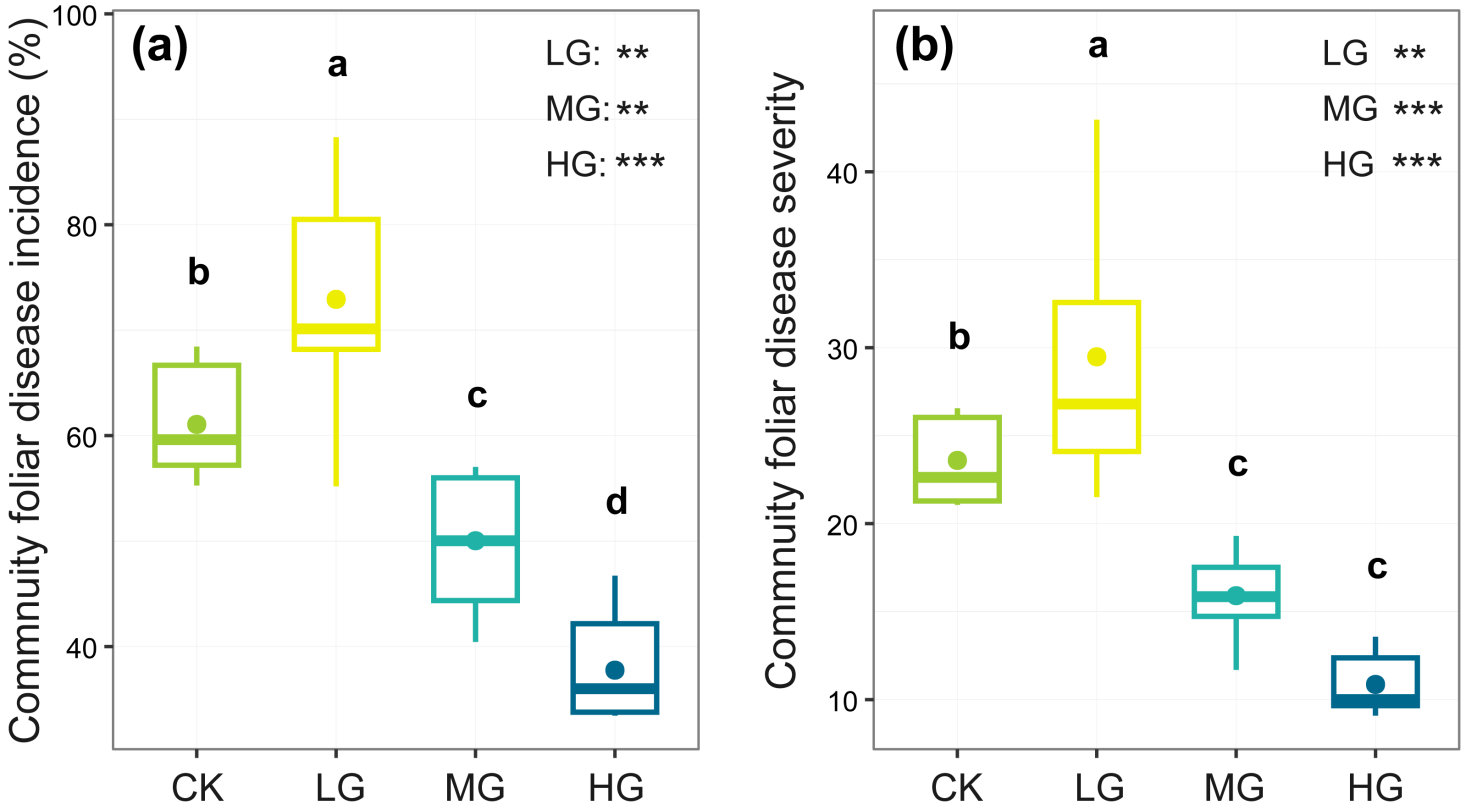
Community foliar disease incidence **(a)** and severity **(b)** across grazing intensity treatments. Boxplots show the 25th and 75th percentiles with the median as a central line; circles indicate the mean, and jittered points represent individual observations. Asterisks denote significant differences from CK based on LMMs (*p < 0.05; **p < 0.01; ***p < 0.001). Different lowercase letters indicate significant differences between treatments (*p* < 0.05, Tukey’s HSD). CK, no grazing; LG, light grazing; MG, moderate grazing; and HG, heavy grazing.

**Figure 2:**
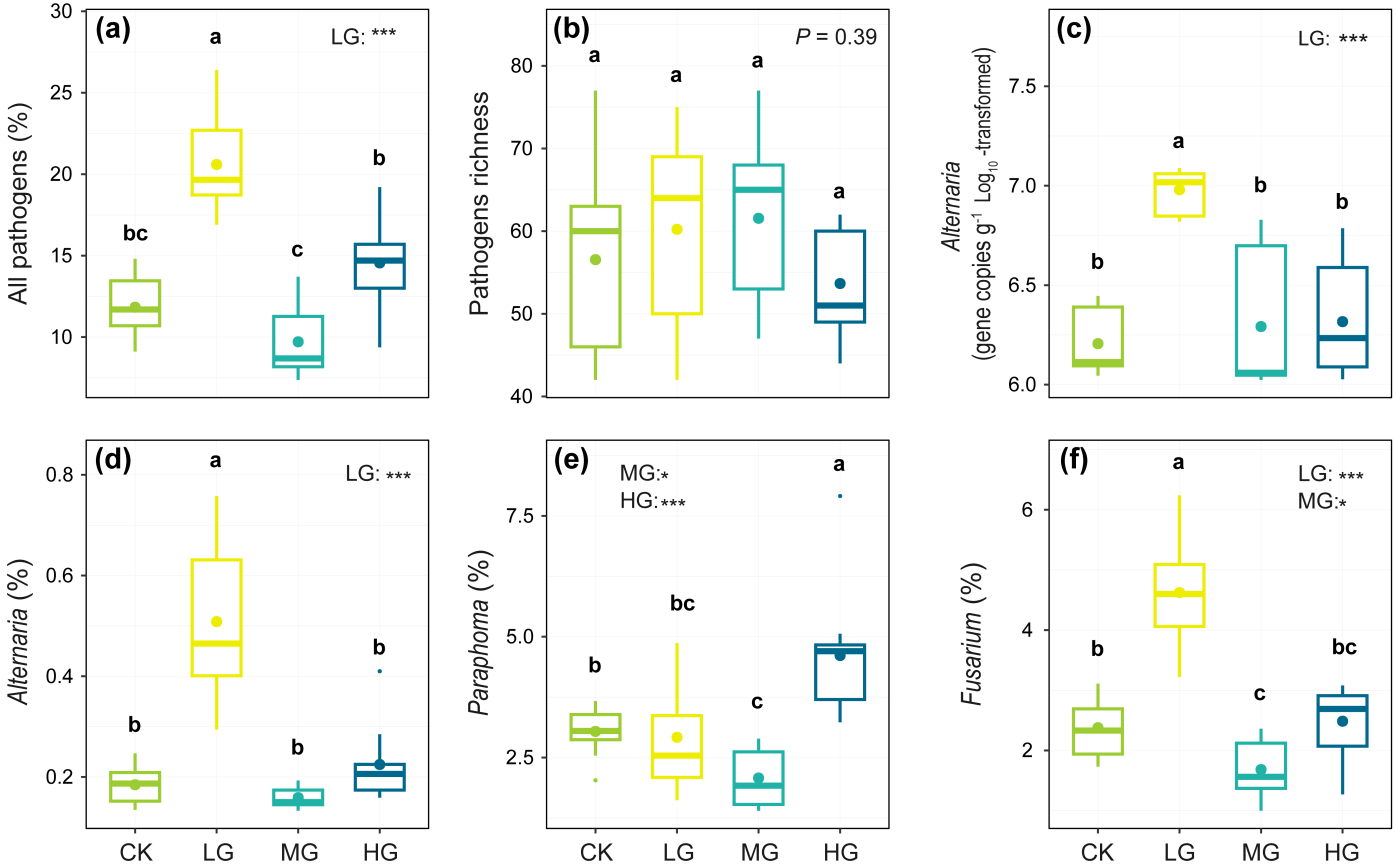
The relative abundance of all soil pathogens **(a)**, pathogen richness **(b)**, absolute abundance of *Alternaria* **(c)** and the relative abundance of *Alternaria* **(d)**, *Paraphoma* **(e)**, and *Fusarium* **(f)** across grazing intensity treatments. Boxplots show the 25th and 75th percentiles with the median as a central line; circles indicate the mean, and jittered points represent individual observations. Asterisks denote significant differences from CK based on LMMs (*p < 0.05; **p < 0.01; ***p < 0.001). Different lowercase letters indicate significant differences between treatments (*p* < 0.05, Tukey’s HSD). CK, no grazing; LG, light grazing; MG, moderate grazing; and HG, heavy grazing.

Light grazing significantly increased community foliar disease incidence and severity, whereas moderate and heavy grazing significantly decreased them, and this reduction was more pronounced under heavy grazing (**Figure 1**; **Supplementary Table 4**). Similarly, light grazing significantly increased the relative abundance of all soil pathogens (**Figure 2a**; **Supplementary Table 4**), the absolute abundance of *Alternaria* (**Figure 2c**; **Supplementary Table 4**), and the relative abundances of *Fusarium* and *Alternaria* (**Figures 2d, f**; **Supplementary Table 4**). Moderate grazing significantly decreased the relative abundance of *Paraphoma* and *Fusarium* (**Figures 2e, f**; **Supplementary Table 4**), while heavy grazing significantly increased the relative abundance of *Paraphoma* (**Figure 2e**; **Supplementary Table 4**).

### 3.3 Soil properties

**Figure 3:**
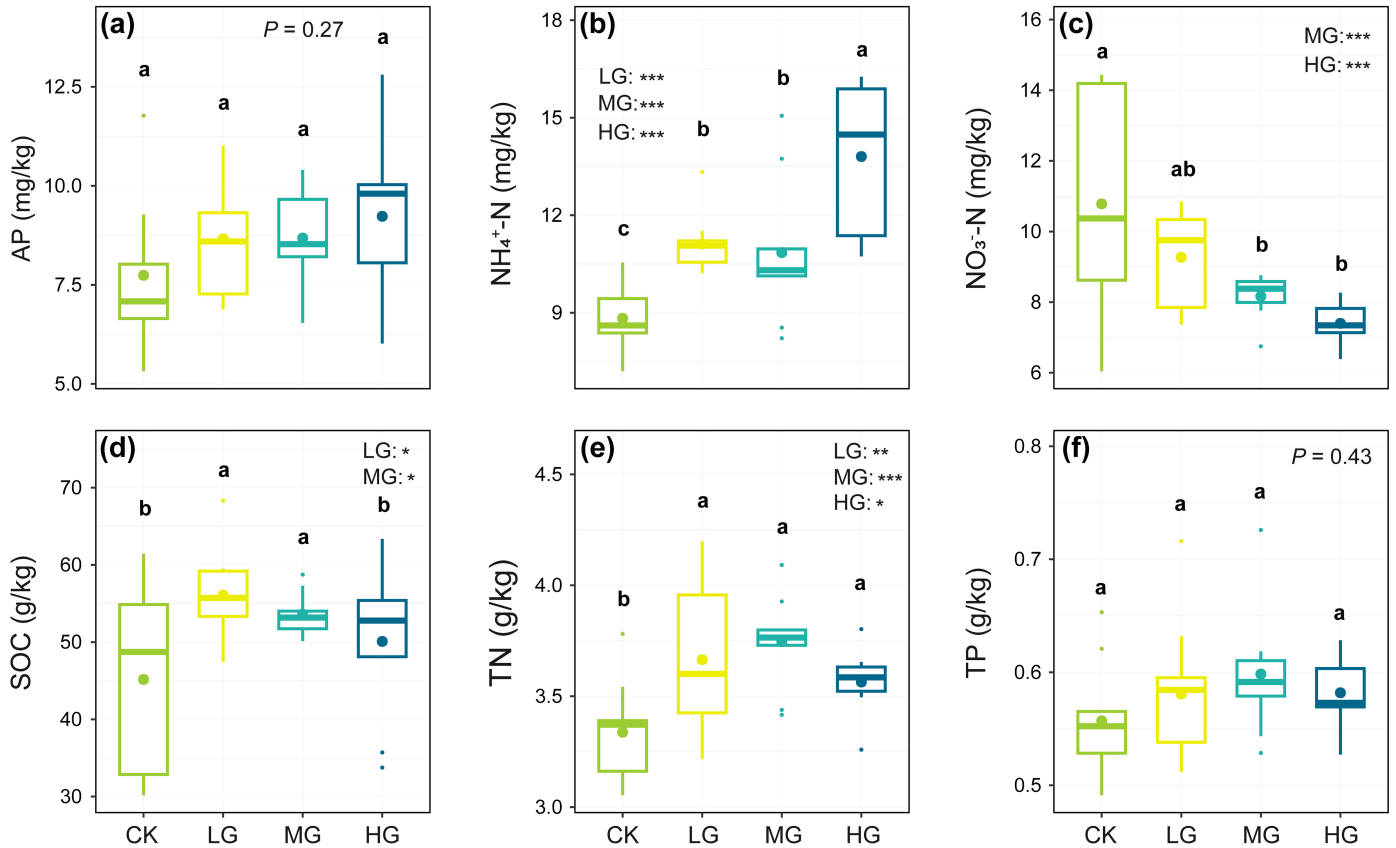
Soil available phosphorus [AP, **(a)**], ammonium nitrogen [NH_4_^+^-N, **(b)**], nitrate nitrogen [NO_3_^--^N, **(c)**], soil organic carbon [SOC, **(d)**], total nitrogen [TN, **(e)**], and total phosphorus [TP, **(f)**] across grazing intensity treatments. Boxplots show the 25th and 75th percentiles with the median as a central line; circles indicate the mean, and jittered points represent individual observations. Asterisks denote significant differences from CK based on LMMs (*p < 0.05; **p < 0.01; ***p < 0.001). Different lowercase letters indicate significant differences between treatments (*p* < 0.05, Tukey’s HSD). CK, no grazing; LG, light grazing; MG, moderate grazing; and HG, heavy grazing.

All grazing intensity treatments significantly increased soil NH_4_^+^-N and TN contents (**Figures 3b, e**; **Supplementary Table 5**). Light and moderate grazing significantly increased soil SOC content (**Figure 3d**; **Supplementary Table 5**), whereas moderate and heavy grazing significantly decreased soil NO_3_^--^N content (**Figure 3c**; **Supplementary Table 5**). Grazing intensity had no significant effects on AP or TP contents (**Figures 3a, f**; **Supplementary Table 5**).

### 3.4 Potential pathways by which grazing intensity affects community foliar diseases

**Figure 4:**
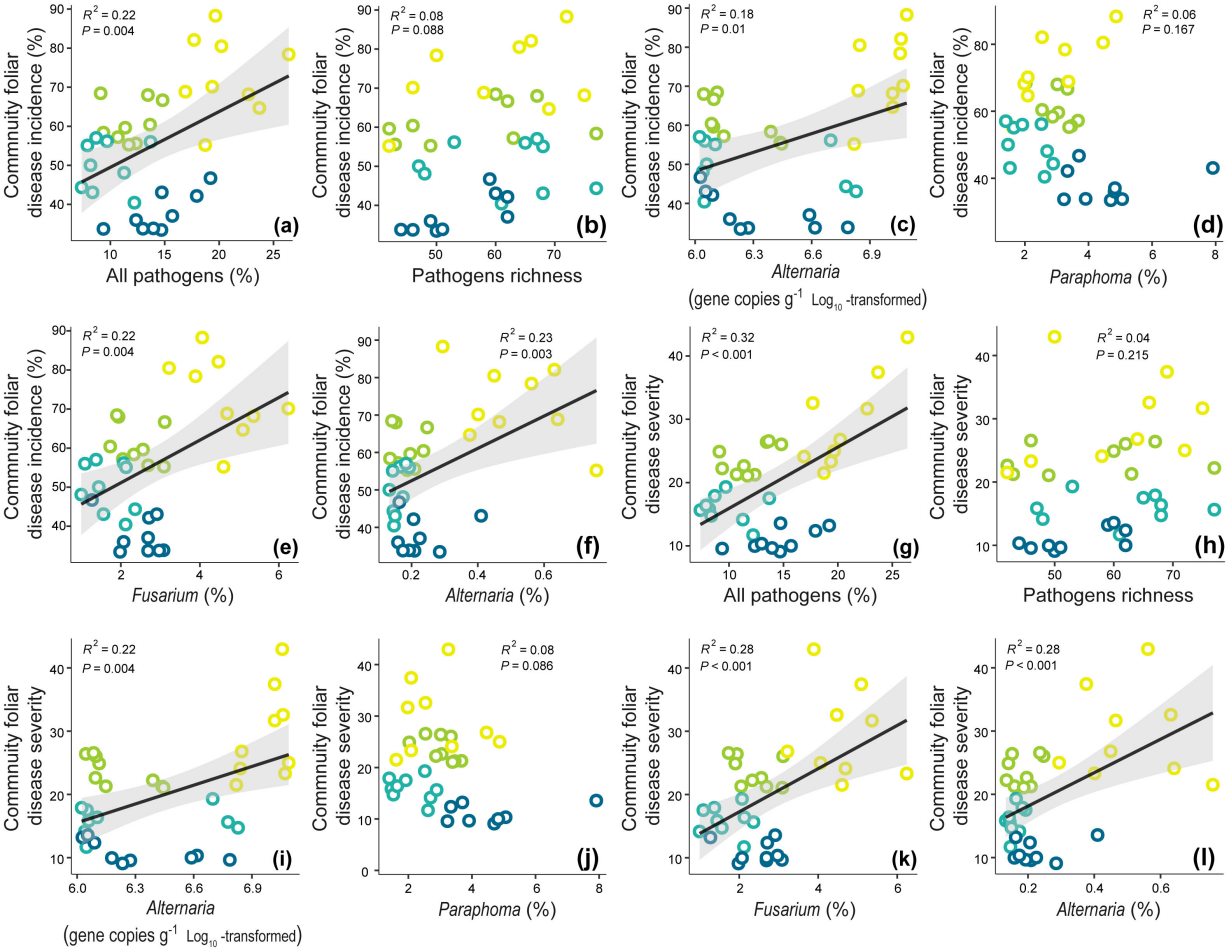
Relationships of community foliar disease incidence **(a-f)** and severity **(g-l)** with the relative abundance of all pathogens **(a, g)**, pathogen richness **(b, h)**, the absolute abundance of *Alternaria* **(c, i)**, and the relative abundance of *Paraphoma* **(d, j)**, *Fusarium* **(e, k)**, and *Alternaria* **(f, l)**. Solid lines and shaded areas represent linear regressions and 95% confidence intervals (*p* < 0.05). *R*^2^ and *p* values are provided in each panel. CK, no grazing; LG, light grazing; MG, moderate grazing; and HG, heavy grazing.

Community foliar disease incidence and severity were significantly and positively correlated with the relative abundance of all soil pathogens (**Figures 4a, g**), the absolute abundance of *Alternaria* (**Figures 4c, i**), and the relative abundances of *Fusarium* and *Alternaria* (**Figures 4e, f, k, l**), whereas they were not significantly correlated with pathogen richness or the relative abundance of *Paraphoma* (**Figures 4b, d, h, j**).

**Figure 5:**
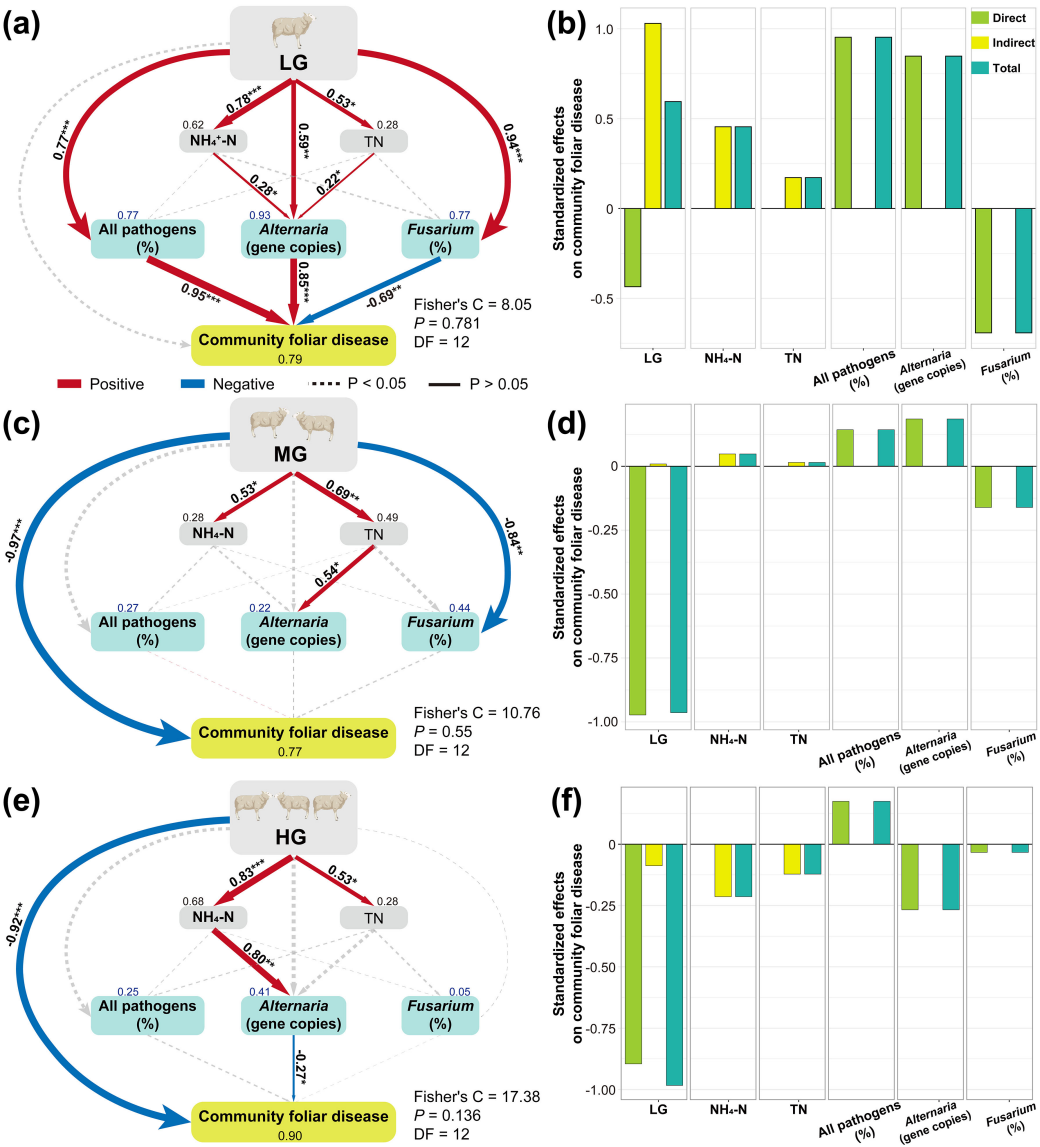
Piecewise structural equation model (SEM) revealed the direct and indirect effects of **(a)** light (LG), **(c)** moderate (MG), and **(e)** heavy grazing (HG) on community foliar disease. For each SEM, red and blue arrows denote significantly positive and negative pathways, respectively. Dashed arrows indicate non-significant connections. The thickness of the arrows indicates the magnitude of the effect. Numbers above variables represent the *R*^2^ (the proportion of variance explained). Asterisks indicate statistically significant corrections: *p < 0.05; **p < 0.01; ***p < 0.001. The summarized direct, indirect, and total effects of drivers on community foliar disease **(b, d, f)**. NH_4_^+^-N, ammonium nitrogen; TN, total nitrogen.

Results from the piecewise SEM revealed distinct pathways through which different grazing intensities regulated community foliar disease via soil properties and soil pathogens (**Figure 5**). Specifically, light grazing not only increased the absolute abundance of *Alternaria* by elevating soil NH_4_^+^-N and TN contents, but also increased the relative abundance of all pathogens, thereby indirectly increasing community foliar disease (**Figures 5a, b**). In contrast, moderate and heavy grazing directly decreased community foliar disease, and this result could not be explained by the indirect effects of soil properties or soil pathogens (**Figures 5c-f**).

## 4 Discussion

Our long-term experiment revealed how grazing intensity influences soil pathogens and foliar disease. Light grazing increased community foliar disease incidence and severity, whereas moderate and heavy grazing reduced them. Furthermore, our findings provide evidence that light grazing increased the relative abundance of all soil pathogens as well as the relative and absolute abundance of dominant genera such as *Alternaria*, and these changes can further exacerbate the prevalence and transmission of foliar diseases. However, the decline in foliar diseases under moderate and heavy grazing could not be explained by changes in soil pathogens. These findings are crucial for managing grassland health in the face of intensifying human activities.

### 4.1 Effects of grazing intensity on soil pathogens

We found that light grazing significantly increased the relative abundance of all soil pathogens and dominant genera (*Alternaria* and *Fusarium*). Consistent with our results, a recent study indicates that grazing significantly increases the proportion of pathogens in colder grasslands (Wang et al., 2024). Generally, grazers can influence soil microbial community dynamics by altering soil nutrient availability (Bardgett and Wardle, 2003; Wang et al., 2020). Light grazing can accelerate soil N mineralization and organic matter decomposition by stimulating root exudation (Shariff et al., 1994; Zhu et al., 2025). These nutrient-rich conditions may, in turn, promote the growth and proliferation of soil fungal pathogens (Lekberg et al., 2021; Singh et al., 2025). In addition, soil pathogens under N-enriched conditions may exhibit stronger niche and resource competition ability than beneficial microorganisms with antagonistic effects, such as *Bacillus* and *Trichoderma* (Zhang et al., 2022; Xie et al., 2026). In our study, light grazing significantly increased soil NH_4_^+^-N, TN and SOC, suggesting that the resulting increase in soil nutrients and resources may be a key driver of the higher abundance of soil pathogens. This is supported by a recent study showing that N enrichment across global grasslands increases the relative abundance of soil fungal pathogens while suppressing mutualistic fungi (Lekberg et al., 2021).

Nevertheless, we found that moderate grazing generally reduced the relative abundance of soil pathogens, while heavy grazing had no significant effect, despite both increasing soil nutrient levels. This contrasts with the patterns observed under light grazing, as moderate and heavy grazing also increased soil nutrient levels (**Figure 3**). These divergent responses suggest that, in addition to soil nutrients, soil pathogens may also be influenced by other driving factors, such as aboveground plant diversity and soil physical properties (Rottstock et al., 2014; Keesing et al., 2006; Beylich et al., 2010). According to the intermediate disturbance hypothesis, moderate grazing is expected to enhance plant diversity by suppressing dominant species and promoting resource redistribution (Grime, 1973; Milchunas et al., 1988). On the one hand, higher plant diversity can foster stable and beneficial microbial communities by providing a wider range of resources and creating a more complex soil microenvironment (Eisenhauer et al., 2010; Lange et al., 2015), thereby diluting or suppressing soil pathogens (Latz et al., 2012; Xiong et al., 2026). On the other hand, diverse root exudates can further suppress soil pathogens by recruiting pathogen-antagonistic microbes, such as *Pseudomonas* (Hu et al., 2018; Wu et al., 2026). In contrast, although increased dung and urine input under heavy grazing enhanced soil nutrient availability, intensive removal of aboveground biomass and trampling reduced plant diversity, resource inputs, and soil moisture, while increasing soil bulk density (Guo et al., 2024; Tian et al., 2026). Such deterioration of soil habitat conditions may offset the potential benefits of increased nutrient availability for soil pathogens, which may explain the neutral effect of heavy grazing on soil pathogens.

### 4.2 Effects of grazing intensity on community foliar diseases

Similarly, our results showed that light grazing increased community foliar disease incidence and severity, consistent with a previous study conducted in alpine grasslands of the Qinghai-Tibetan Plateau (Mipam et al., 2022). Furthermore, our SEM results demonstrate that light grazing indirectly exacerbated community foliar disease by increasing the abundance of all soil pathogens and *Alternaria* (**Figures 5a, b**). On the one hand, this may be related to the disease type. According to pathogen life history strategies, foliar diseases can be classified into biotrophic diseases (e.g. rusts and powdery mildews) and necrotrophic diseases (e.g. leaf spot). Biotrophic pathogens survive only in living plant tissues, and their spores may be directly removed by grazers (Thaler et al., 2010). Conversely, necrotrophic pathogens can persist in diverse environments, including soil and plant litter (Liu et al., 2021, 2022). In our study system, foliar diseases were predominantly leaf spot diseases, suggesting that soil pathogens are the primary source of foliar infection (**Supplementary Table 1**). More importantly, we found that foliar disease was significantly and positively correlated with the relative abundance of all soil pathogens and the absolute abundance of dominant pathogens (**Figure 4**). Thus, the increase in community foliar disease under light grazing may be associated with the higher abundance of soil pathogens, particularly *Alternaria*. Interestingly, we found that the two species with the highest foliar disease incidence and severity (*C. aridula* and *K. humilis*) were both infected by *Alternaria* (**Supplementary Figure 1**), and foliar disease in these species also increased under light grazing, further supporting our speculation (**Supplementary Tables 1**, **2**). Furthermore, damage inflicted by herbivores can create entry points for pathogen infection. Compared to biotrophic pathogens, necrotrophic pathogens, which are abundant and diverse, may benefit more from the wounds caused to plant tissue by grazers (Daleo et al., 2009; Stout et al., 2006). Our study quantitatively distinguished soil pathogens from foliar diseases under grazing and linked them using path analysis, providing a novel perspective that incorporating soil pathogen variables helps explain the mechanisms underlying grazing effects on foliar fungal diseases.

Conversely, we found that moderate and heavy grazing suppressed community foliar disease through direct rather than indirect pathways (**Figures 5c, d–f**). This was likely driven by high-intensity defoliation caused by grazers. In our study, aboveground defoliation rates under both moderate and heavy grazing were set above 50%. In addition, *C. aridula* and *K. humilis* have been shown to be two highly palatable sedge species, and livestock can consume the whole aboveground biomass of these species (Tian et al., 2023). This suggests that although leaf tissue wounds may facilitate pathogen infection, the removal of diseased aboveground tissues under high-intensity defoliation likely outweighs this facilitative effect, thereby suppressing community foliar disease. In support of this interpretation, we found that both population and community-level foliar disease incidence and severity were further significantly reduced under heavy grazing compared with moderate grazing (**Figure 1**; **Supplementary Tables 2**, **3**). Another possible explanation is the plant growth-defense trade-off (Moreira et al., 2018; Mertens et al., 2021). Frequent and intensive herbivory may reduce plant investment in growth while activating defense responses, thereby alleviating foliar disease (Fernández de Bobadilla et al., 2022; Hu et al., 2025). A study on alfalfa provides supporting evidence, showing that aphid feeding activated the jasmonic acid signaling pathway and induced the production of defensive secondary metabolites, ultimately reducing pathogen infection (Wang et al., 2025).

### 4.3 Limitations and perspectives

Although our study clearly assessed the responses of soil pathogens and foliar diseases to grazing intensity in grassland ecosystems, several limitations should be acknowledged. First, plant community characteristics such as species diversity and vegetation cover may also represent key drivers of soil pathogen and foliar disease infection and prevalence (Makiola et al., 2022; Wang et al., 2024). For example, the dilution effect suggests that increasing host plant species diversity can reduce pathogen transmission by decreasing host contact rates (Keesing et al., 2006; Johnson et al., 2015). This interpretation is supported by a long-term grazing experiment conducted in alpine grasslands, which emphasized that community Pielou’s evenness is an important predictor of changes in community foliar disease (Mipam et al., 2022). As a consequence, more plant community characteristics should be included in future studies on how grazing affects plant pathogens to improve our understanding of disease dynamics and develop a multi-factor predictive framework for grassland disease. Second, microenvironmental conditions may also differentially affect pathogen dynamics (Eastburn et al., 2011). For example, high temperature generally promotes pathogen spore production and hyphal growth, while low humidity has the opposite effect (Huber and Gillespie, 1992; Harvell et al., 2002; Siebold and von Tiedemann, 2013). Grazing can alter soil and plant community microenvironments, but how these changes affect soil and foliar pathogens remains an open question. In addition, our study was conducted during a single peak growing season (August), and therefore does not capture seasonal variation in foliar disease dynamics. Future studies should incorporate multi-season sampling to better resolve temporal patterns of disease development.

## 5 Conclusion

Our findings highlight that different grazing intensities have strong and contrasting effects on soil pathogens and foliar diseases in grassland ecosystems. More importantly, we provide new evidence that the occurrence and prevalence of foliar diseases are closely related to soil pathogen abundance. Livestock abundance and density are changing due to intensified human activities, and these changes can regulate soil pathogens and foliar diseases in grassland ecosystems. Our study suggests that light grazing is likely to increase the risk of grassland plant pathogen outbreaks, whereas moderate grazing reduces it. Overall, these findings not only advance our understanding of disease ecology but also provide novel insights into plant disease management in alpine grassland grazing systems.

## Data Availability

The original contributions presented in the study are included in the article/**Supplementary Material**. Further inquiries can be directed to the corresponding author.

## References

[B1] Allan E. Van Ruijven J. Crawley M. J. (2010). Foliar fungal pathogens and grassland biodiversity. Ecology 91, 2572–2582. doi: 10.1890/09-0859 20957952

[B2] Bai Y. Cotrufo M. F. (2022). Grassland soil carbon sequestration: Current understanding, challenges, and solutions. Science 377, 603–608. doi: 10.1126/science.abo2380 35926033

[B3] Bardgett R. D. Wardle D. A. (2003). Herbivore‐mediated linkages between aboveground and belowground communities. Ecology 84, 2258–2268. doi: 10.1890/02-0274

[B4] Beylich A. Oberholzer H. R. Schrader S. Höper H. Wilke B. M. (2010). Evaluation of soil compaction effects on soil biota and soil biological processes in soils. Soil Tillage Res. 109, 133–143. doi: 10.1016/j.still.2010.05.010 38826717

[B5] Borer E. T. Mitchell C. E. Power A. G. Seabloom E. W. (2009). Consumers indirectly increase infection risk in grassland food webs. Proc. Natl. Acad. Sci. U.S.A. 106, 503–506. doi: 10.1073/pnas.0808778106 19126681 PMC2626732

[B6] Callahan B. J. McMurdie P. J. Rosen M. J. Han A. W. Johnson A. J. Holmes S. P. (2016). DADA2: High-resolution sample inference from Illumina amplicon data. Nat. Methods 13, 581–583. doi: 10.1038/nmeth.3869 27214047 PMC4927377

[B7] Cappelli S. L. Pichon N. A. Kempel A. Allan E. (2020). Sick plants in grassland communities: a growth‐defense trade‐off is the main driver of fungal pathogen abundance. Ecol. Lett. 23, 1349–1359. doi: 10.1111/ele.13537 32455502

[B8] Case N. T. Gurr S. J. Fisher M. C. Blehert D. S. Boone C. Casadevall A. . (2025). Fungal impacts on Earth’s ecosystems. Nature 638, 49–57. doi: 10.1038/s41586-024-08419-4 39910383 PMC11970531

[B9] Daleo P. Silliman B. Alberti J. Escapa M. Canepuccia A. Peña N. . (2009). Grazer facilitation of fungal infection and the control of plant growth in south-western Atlantic salt marshes. J. Ecol. 97, 781–787. doi: 10.1111/j.1365-2745.2009.01508.x 40046247

[B10] Delgado-Baquerizo M. Guerra C. A. Cano-Díaz C. Egidi E. Wang J. T. Eisenhauer N. . (2020). The proportion of soil-borne pathogens increases with warming at the global scale. Nat. Clim. Change 10, 550–554. doi: 10.1038/s41558-020-0759-3 37880705

[B11] Du S. Trivedi P. Wei Z. Feng J. Hu H. W. Bi L. . (2022). The proportion of soil-borne fungal pathogens increases with elevated organic carbon in agricultural soils. Msystems 7, e01337–e01321. doi: 10.1128/msystems.01337-21 35311561 PMC9040864

[B12] Duan D. Tian Z. Wu N. Feng X. Hou F. Nan Z. . (2023). Drought neutralizes positive effects of long‐term grazing on grassland productivity through altering plant–soil interactions. Funct. Ecol. 37, 1827–1840. doi: 10.1111/1365-2435.14346 40046247

[B13] Eastburn D. M. McElrone A. J. Bilgin D. D. (2011). Influence of atmospheric and climatic change on plant–pathogen interactions. Plant Pathol. 60, 54–69. doi: 10.1111/j.1365-3059.2010.02402.x 40046247

[B14] Eisenhauer N. Beßler H. Engels C. Gleixner G. Habekost M. Milcu A. . (2010). Plant diversity effects on soil microorganisms support the singular hypothesis. Ecology 91, 485–496. doi: 10.1890/08-2338.1 20392013

[B15] Fernández de Bobadilla M. Vitiello A. Erb M. Poelman E. H. (2022). Plant defense strategies against attack by multiple herbivores. Trends Plant Sci. 27, 528–535. doi: 10.1016/j.tplants.2021.12.010 35027280

[B16] Fisher M. C. Henk D. A. Briggs C. J. Brownstein J. S. Madoff L. C. McCraw S. L. . (2012). Emerging fungal threats to animal, plant and ecosystem health. Nature 484, 186–194. doi: 10.1038/nature10947 22498624 PMC3821985

[B17] Grime J. P. (1973). Competitive exclusion in herbaceous vegetation. Nature 242, 344–347. doi: 10.1038/242344a0 37880705

[B18] Guo T. Guo M. Pang Y. Sun X. Ryo M. Liu N. . (2024). Ungulate herbivores promote beta diversity and drive stochastic plant community assembly by selective defoliation and trampling: From a four‐year simulation experiment. J. Ecol. 112, 1992–2006. doi: 10.1111/1365-2745.14370 40046247

[B19] Harvell C. D. Mitchell C. E. Ward J. R. Altizer S. Dobson A. P. Ostfeld R. S. . (2002). Climate warming and disease risks for terrestrial and marine biota. Science 296, 2158–2162. doi: 10.1126/science.1063699 12077394

[B20] Herrero‐Jáuregui C. Oesterheld M. (2018). Effects of grazing intensity on plant richness and diversity: A meta‐analysis. Oikos 127, 757–766. doi: 10.1111/oik.04893 40046247

[B21] Hu L. Robert C. A. Cadot S. Zhang X. I. Ye M. Li B. . (2018). Root exudate metabolites drive plant-soil feedbacks on growth and defense by shaping the rhizosphere microbiota. Nat. Commun. 9, 2738. doi: 10.1038/s41467-018-05122-7 30013066 PMC6048113

[B22] Hu L. Zhang K. Xu Y. Zheng X. Waterman J. M. Ouyang X. . (2025). Herbivory-induced green leaf volatiles increase plant performance through jasmonate-dependent plant–soil feedbacks. Nat. Plants 11, 1001–1017. doi: 10.1038/s41477-025-01987-x 40312547

[B23] Huber D. M. Watson R. D. (1974). Nitrogen form and plant disease. Annu. Rev. Phytopathol. 12, 139–157. doi: 10.1146/annurev.py.12.090174.001035 23249125

[B24] Huber L. Gillespie T. J. (1992). Modeling leaf wetness in relation to plant disease epidemiology. Annu. Rev. Phytopathol. 30, 553–577. doi: 10.1146/annurev.py.30.090192.003005 41139587

[B25] Johnson P. T. Ostfeld R. S. Keesing F. (2015). Frontiers in research on biodiversity and disease. Ecol. Lett. 18, 1119–1133. doi: 10.1111/ele.12479 26261049 PMC4860816

[B26] Keesing F. Holt R. D. Ostfeld R. S. (2006). Effects of species diversity on disease risk. Ecol. Lett. 9, 485–498. doi: 10.1111/j.1461-0248.2006.00885.x 16623733

[B27] Lange M. Eisenhauer N. Sierra C. A. Bessler H. Engels C. Griffiths R. I. . (2015). Plant diversity increases soil microbial activity and soil carbon storage. Nat. Commun. 6, 6707. doi: 10.1038/ncomms7707 25848862

[B28] Latz E. Eisenhauer N. Rall B. C. Allan E. Roscher C. Scheu S. . (2012). Plant diversity improves protection against soil‐borne pathogens by fostering antagonistic bacterial communities. J. Ecol. 100, 597–604. doi: 10.1111/j.1365-2745.2011.01940.x 40046247

[B29] Lekberg Y. Arnillas C. A. Borer E. T. Bullington L. S. Fierer N. Kennedy P. G. . (2021). Nitrogen and phosphorus fertilization consistently favor pathogenic over mutualistic fungi in grassland soils. Nat. Commun. 12, 3484. doi: 10.1038/s41467-021-23605-y 34108462 PMC8190096

[B30] Li S. Li K. Zhang T. Tang S. Zhang Z. Chen H. . (2025). The global effects of grazing on grassland soil nitrogen retention. Agric. Ecosyst. Environ. 383, 109516. doi: 10.1016/j.agee.2025.109516 38826717

[B31] Li P. Tedersoo L. Crowther T. W. Wang B. Shi Y. Kuang L. . (2023). Global diversity and biogeography of potential phytopathogenic fungi in a changing world. Nat. Commun. 14, 6482. doi: 10.1038/s41467-023-42142-4 37838711 PMC10576792

[B32] Liu Y. Duan D. Jiang F. Tian Z. Feng X. Wu N. . (2022). Long‐term heavy grazing increases community‐level foliar fungal diseases by shifting plant composition. J. Appl. Ecol. 59, 791–800. doi: 10.1111/1365-2664.14093 40046247

[B33] Liu M. Mipam T. D. Wang X. Zhang P. Lin Z. Liu X. (2021). Contrasting effects of mammal grazing on foliar fungal diseases: patterns and potential mechanisms. New Phytol. 232, 345–355. doi: 10.1111/nph.17324 33666239

[B34] Liu Y. Zhao X. Liu W. Yang X. Feng B. Zhang C. . (2023). Herbivore assemblages affect soil microbial communities by altering root biomass and available nutrients in an alpine meadow. Front. Plant Sci. 14, 1117372. doi: 10.3389/fpls.2023.1117372 36938013 PMC10017739

[B35] Makiola A. Holdaway R. J. Wood J. R. Orwin K. H. Glare T. R. Dickie I. A. (2022). Environmental and plant community drivers of plant pathogen composition and richness. New Phytol. 233, 496–504. doi: 10.1111/nph.17797 34651304

[B36] Mertens D. Fernández de Bobadilla M. Rusman Q. Bloem J. Douma J. C. Poelman E. H. (2021). Plant defence to sequential attack is adapted to prevalent herbivores. Nat. Plants 7, 1347–1353. doi: 10.1038/s41477-021-00999-7 34650263

[B37] Milchunas D. G. Sala O. E. Lauenroth W. K. (1988). A generalized model of the effects of grazing by large herbivores on grassland community structure. Am. Nat. 132, 87–106. doi: 10.1086/284839 37306075

[B38] Mipam T. D. Chen F. Tian L. Zhang P. Huang M. Chen L. . (2022). Plant community-mediated effects of grazing on plant diseases. Oecologia 199, 897–905. doi: 10.1007/s00442-022-05223-7 35907123

[B39] Mitchell C. E. Reich P. B. Tilman D. Groth J. V. (2003). Effects of elevated CO2, nitrogen deposition, and decreased species diversity on foliar fungal plant disease. Glob. Change Biol. 9, 438–451. doi: 10.1046/j.1365-2486.2003.00602.x 37945311

[B40] Moreira X. Abdala‐Roberts L. Castagneyrol B. (2018). Interactions between plant defence signalling pathways: evidence from bioassays with insect herbivores and plant pathogens. J. Ecol. 106, 2353–2364. doi: 10.1111/1365-2745.12987 40046247

[B41] Nguyen N. H. Song Z. Bates S. T. Branco S. Tedersoo L. Menke J. . (2016). FUNGuild: an open annotation tool for parsing fungal community datasets by ecological guild. Fungal Ecol. 20, 241–248. doi: 10.1016/j.funeco.2015.06.006 38826717

[B42] Parker I. M. Saunders M. Bontrager M. Weitz A. P. Hendricks R. Magarey R. . (2015). Phylogenetic structure and host abundance drive disease pressure in communities. Nature 520, 542–547. doi: 10.1038/nature14372 25903634

[B43] Parry D. W. Jenkinson P. McLeod L. (1995). Fusarium ear blight (scab) in small grain cereals—a review. Plant Pathol. 44, 207–238. doi: 10.1111/j.1365-3059.1995.tb02773.x

[B44] Peng Z. Liu Y. Qi J. Gao H. Li X. Tian Q. . (2023). The climate‐driven distribution and response to global change of soil‐borne pathogens in agroecosystems. Glob. Ecol. Biogeogr. 32, 766–779. doi: 10.1111/geb.13662 40046247

[B45] Pichon B. Kéfi S. Gounand I. Gross N. Le Bagousse‐Pinguet Y. Guerber J. . (2025). Grazing modulates the multiscale spatial structure of dryland vegetation. Glob. Change Biol. 31, e70345. doi: 10.1111/gcb.70345 40654195 PMC12257447

[B46] Pillar V. D. Winck B. R. (2026). Natural grasslands used for grazing livestock can mitigate climate change. Science 391, eaea8344. doi: 10.1126/science.aea8344 41505549

[B47] R Core Team (2024). R: A Language and Environment for Statistical Computing (Vienna, Austria: R Foundation for Statistical Computing). Available online at: https://www.R-project.org (Accessed May 28, 2026).

[B48] Rottstock T. Joshi J. Kummer V. Fischer M. (2014). Higher plant diversity promotes higher diversity of fungal pathogens, while it decreases pathogen infection per plant. Ecology 95, 1907–1917. doi: 10.1890/13-2317.1 25163123

[B49] Shariff A. R. Biondini M. E. Grygiel C. E. (1994). Grazing intensity effects on litter decomposition and soil nitrogen mineralization. J. Range Manage. 47, 444–449. doi: 10.2307/4002994

[B50] Siebold M. von Tiedemann A. (2013). Effects of experimental warming on fungal disease progress in oilseed rape. Glob. Change Biol. 19, 1736–1747. doi: 10.1111/gcb.12180 23505251

[B51] Singh B. K. Delgado-Baquerizo M. Egidi E. Guirado E. Leach J. E. Liu H. . (2023). Climate change impacts on plant pathogens, food security and paths forward. Nat. Rev. Microbiol. 21, 640–656. doi: 10.1038/s41579-023-00900-7 37131070 PMC10153038

[B52] Singh B. K. Jiang G. Wei Z. Sáez-Sandino T. Gao M. Liu H. . (2025). Plant pathogens, microbiomes, and soil health. Trends Microbiol. 33, 887–902. doi: 10.1016/j.tim.2025.03.013 40274492

[B53] Stout M. J. Thaler J. S. Thomma B. P. (2006). Plant-mediated interactions between pathogenic microorganisms and herbivorous arthropods. Annu. Rev. Entomol. 51, 663–689. doi: 10.1146/annurev.ento.51.110104.151117 16332227

[B54] Thaler J. S. Agrawal A. A. Halitschke R. (2010). Salicylate‐mediated interactions between pathogens and herbivores. Ecology 91, 1075–1082. doi: 10.1890/08-2347.1 20462121

[B55] Tian Z. Feng X. Wu N. Liu H. Dong X. Zhang Y. . (2026). Context-dependent responses of soil microbes and nematodes to grazing: Evidence from a global meta-analysis. J. Appl. Ecol. 63, e70405. doi: 10.1111/1365-2664.70405 40046247

[B56] Tian Z. Li W. Kou Y. Dong X. Liu H. Yang X. . (2023). Effects of different livestock grazing on foliar fungal diseases in an alpine grassland on the Qinghai–Tibet Plateau. J. Fungi 9, 949. doi: 10.3390/jof9090949 37755057 PMC10533196

[B57] Veresoglou S. D. Barto E. K. Menexes G. Rillig M. C. (2013). Fertilization affects severity of disease caused by fungal plant pathogens. Plant Pathol. 62, 961–969. doi: 10.1111/ppa.12014 40046247

[B58] Wang T. T. Li Y. Z. (2025). First report of Nectriella adonidis causing root rot of Lespedeza chinensis in China. Plant Dis. 109, 2607. doi: 10.1094/PDIS-07-25-1389-PDN

[B59] Wang Y. Tian Z. Li Y. Nan Z. Duan T. (2025). Arbuscular mycorrhizal fungus alters the phyllosphere microbial community and modifies plant defences against co‐attack of pea aphid and pathogen. Funct. Ecol. 39, 3181–3196. doi: 10.1111/1365-2435.70169 40046247

[B60] Wang B. Wu L. Chen D. Wu Y. Hu S. Li L. . (2020). Grazing simplifies soil micro‐food webs and decouples their relationships with ecosystem functions in grasslands. Glob. Change Biol. 26, 960–970. doi: 10.1111/gcb.14841 31529564

[B61] Wang Y. Zhang M. Delgado-Baquerizo M. Li G. Cai J. Pan X. . (2024). Long-term grazing effects on soil-borne pathogens are driven by temperature. Commun. Biol. 7, 1568. doi: 10.1038/s42003-024-07280-5 39587336 PMC11589589

[B62] White T. J. Bruns T. D. Lee S. B. Taylor J. W. (1990). “ Amplification and direct sequencing of fungal ribosomal RNA genes for phylogenetics,” in PCR Protocols: A Guide to Methods and Applications, vol. 18 . Eds. Innis M. A. Gelfand D. H. Sninsky J. J. White T. J. ( Academic Press, New York), 315–322.

[B63] Wu C. Liu H. Carvalhais L. C. Guo J. Cai P. Zhong J. . (2026). Root exudate‐associated microbiome assembly contributes to viral disease resistance in wheat. New Phytol. 251, 1397–1414. doi: 10.1111/nph.71254 42136370

[B64] Xie J. Sun X. Wen T. Bai Y. Qian T. Hu S. . (2026). Metabolite interactions mediate beneficial alliances between *Bacillus* and *Trichoderma* for effective Fusarium wilt control. ISME J. 20, wraf283. doi: 10.1093/ismejo/wraf283 41454779 PMC12887306

[B65] Xiong C. Ge A. H. Gao M. Singh B. K. (2026). Impacts of climate extremes on plant pathogens, microbiomes and plant health. Nat. Rev. Microbiol., 1–17. doi: 10.1038/s41579-026-01290-2 41814018

[B66] Yang X. Dong Q. Chu H. Ding C. Yu Y. Zhang C. . (2019). Different responses of soil element contents and their stoichiometry (C: N: P) to yak grazing and Tibetan sheep grazing in an alpine grassland on the eastern Qinghai–Tibetan Plateau. Agric. Ecosyst. Environ. 285, 106628. doi: 10.1016/j.agee.2019.106628 38826717

[B67] Zhang Y. Ye C. Su Y. Peng W. Lu R. Liu Y. . (2022). Soil acidification caused by excessive application of nitrogen fertilizer aggravates soil-borne diseases: Evidence from literature review and field trials. Agric. Ecosyst. Environ. 340, 108176. doi: 10.1016/j.agee.2022.108176 38826717

[B68] Zhou G. Zhou X. He Y. Shao J. Hu Z. Liu R. . (2017). Grazing intensity significantly affects belowground carbon and nitrogen cycling in grassland ecosystems: A meta‐analysis. Glob. Change Biol. 23, 1167–1179. doi: 10.1111/gcb.13431 27416555

[B69] Zhu A. Wu Q. Han G. (2025). Light grazing enhances microbial-mediated nitrogen transformation in desert steppe soils. BMC Plant Biol. 25, 1301. doi: 10.1186/s12870-025-07356-2 41044767 PMC12495815

