# Supplementary material for: Effects of grazing intensity on plant community fungal diseases in an alpine grassland
Source: Front Plant Sci. 2026 Aug 3;17:1900475. doi: 10.3389/fpls.2026.1900475 (PMC13478247; doi:10.3389/fpls.2026.1900475)
Supplement: Supplementary file 1 [file DataSheet1.docx]

**Supporting Information for**

**Effects of grazing intensity on plant community fungal diseases in an alpine grassland**

**This file includes:**

Methods S1- S3

Tables S1 to S5

Figure S1 to S2

**Methods S1**

***Soil fungal amplicon sequencing***

Genomic DNA was extracted from 0.25 g of each soil sample using the DNeasy PowerSoil Kit (Qiagen, Germany) according to the manufacturer’s instructions. DNA concentration and purity were measured using a NanoDrop One spectrophotometer (Thermo Fisher Scientific, USA) prior to downstream analyses.

The fungal ITS2 region was amplified using the universal fungal primers ITS3 and ITS4 (White et al., 1990). PCR amplification was performed in a total reaction volume of 50 μl containing 25 μl of 2× Premix Taq, 1 μl of forward primer (10 μM), 1 μl of reverse primer (10 μM), 50 ng of template DNA, and nuclease-free water to a final volume of 50 μl. Reactions were carried out on a Bio-Rad S1000 thermal cycler (Bio-Rad Laboratories, CA, USA) under the following conditions: initial denaturation at 94°C for 5 min; followed by 30 cycles of denaturation at 94°C for 30 s, annealing at 52°C for 30 s, and extension at 72°C for 30 s; followed by a final extension at 72°C for 10 min and holding at 4°C. Purified amplicons were used for library preparation and sequenced on an Illumina MiSeq platform (2 × 250 bp paired-end; Illumina Inc., USA).

Raw sequencing reads were demultiplexed and processed in the R environment using the DADA2 pipeline. Sequence processing included quality filtering, denoising, paired-end read merging, and chimera removal. High-resolution amplicon sequence variants (ASVs) were inferred to represent unique fungal sequence types. ASVs with fewer than 5 reads across the full dataset were removed to minimize potential sequencing artifacts and spurious rare variants. The final curated ASV table was used for downstream analyses of soil fungal community composition, diversity, and relative abundance.

.

**Methods S2**

**qPCR analyses**

Total genomic DNA was extracted from 0.25 g of soil using a commercial soil DNA extraction kit following standard protocols. DNA concentration and purity were assessed using a NanoDrop 2000 spectrophotometer (Thermo Fisher Scientific, USA).

The absolute abundance of Alternaria was quantified using SYBR Green I-based real-time quantitative PCR (qPCR) on an ABI 7300 Real-Time PCR System (Applied Biosystems, USA). Primers Dir1ITSAltF (5′-TGTCTTTTGCGTACTTCTTGTTTCCT-3′) and Inv1ITSAltR (5′-CGACTTGTGCTGCGCTC-3′) were used to amplify the ITS2 region of Alternaria spp.

Each 20 μl qPCR reaction contained 10 μl of 2× ChamQ SYBR Color qPCR Master Mix (Vazyme, China), 0.8 μl of each primer (5 μM), 0.4 μl ROX reference dye, 2 μl of template DNA, and 6 μl of nuclease-free water. The thermal cycling conditions were: 95°C for 3 min, followed by 40 cycles of 95°C for 5 s, 58°C for 30 s, and 72°C for 1 min. Melting curve analysis was performed to confirm amplification specificity.

Standard curves were generated using tenfold serial dilutions (10⁻²-10⁻⁸) of plasmid DNA containing the target fragment. Gene copy numbers in soil samples were quantified by comparing Ct values with the standard curves. Amplification efficiency was 92.04%, and the coefficient of determination (*R²*) was 0.998.

**Table S1.** List of host species, their plant functional groups, observed diseases and associated pathogens, and the corresponding mean disease incidence (%) and severity (mean ± standard error) for each species. Notes: * = the standard error (SE) could not be calculated for this value due to insufficient degrees of freedom.

| **Species** | **Plant** **functional group** | **Disease group(s)** | **Pathogen** | **Mean disease incidence (%)** | **Mean disease severity** |
| --- | --- | --- | --- | --- | --- |
| *Ajuga lupulina* | Forb | Leaf spot | Undentified | 18.18* | 3.64* |
| *Aster altaicus* | Forb | Leaf spot | Undentified | 17.26 ± 3.32 | 4.73 ± 1.13 |
| *Astragalus laxmannii* | Forb | Leaf spot | Undentified | 6.67* | 0.62 ±0.62 |
| *Bupleurum chinense* | Forb | Leaf spot | *Septoria* sp. | 76* | 29.60* |
| *Carex aridula* | Cyperaceae | Leaf spot | *Alternaria* sp. | 82.11 ± 2.65 | 30.34 ± 1.83 |
| *Dracocephalum heterophyllum* | Forb | Leaf spot | *Alternaria solani* | 29.35 ± 3.10 | 8.88 ± 1.13 |
| *Elymus nutans* | Grass | Leaf spot | *Parastagonospora nodorum* | 52.00 | 18.40* |
| *Kobresia humilis* | Cyperaceae | Leaf spot | *Alternaria alternata* | 78.67 ± 2.45 | 30.33 ± 1.76 |
| *Koeleria cristata* | Grass | Leaf spot | Undentified | 62.33 ± 3.42 | 22.25 ± 1.51 |
| *Leymus secalinus* | Legume | Leaf spot | *Coniochaeta discospora* | 54.12 ± 5.25 | 19.09 ± 2.37 |
| *Medicago ruthenica* | Legume | Leaf spot | Undentified | 17.78 ± 6.09 | 5.87 ± 2.50 |
| *Oxytropis subfalcata* | Legume | Leaf spot | Undentified | 2.00 ± 2.00 | 0.40 ± 0.40 |
| *Poa annua* | Grass | Leaf spot | *Phoma* sp. | 53.38 ± 2.65 | 17.06 ± 1.09 |
| *Potentilla acaulis* | Forb | Leaf spot | Undentified | 50.00 ± 2.76 | 19.46 ± 1.39 |
| *Potentilla bifurca* | Forb | Leaf spot | *Coniochaeta* sp. | 53.10 ± 5.64 | 17.22 ± 3.10 |
| *Potentilla multicaulis* | Forb | Leaf spot | *Stagonospora* sp*.* | 45.35 ± 4.54 | 15.99 ± 1.76 |
| *Sibbaldia adpressa* | Forb | Leaf spot | *Leptotrochila* sp. | 46.81 ± 5.61 | 15.16 ± 1.94 |
| *Stellera chamaejasme* | Forb | Leaf spot | *Acrophialophora jodhpurensis* | 55.20 ± 4.96 | 17.59 ± 1.88 |
| *Stipa tianschanica* | Grass | Leaf spot | *Septora* sp. | 58.33 ± 4.12 | 19.51 ± 1.63 |
| *Thalictrum aquilegiifolium* | Forb | Leaf spot | *Alternaria alternata* | 71.67 ± 8.33 | 21.07 ± 1.87 |
| *Thermopsis lanceolata* | Legume | Leaf spot | *Cercospora* sp. | 26* | 7.2* |

**Table S2.** Mean ± standard error (SE) of the disease incidence (%) for each species under control (CK), light grazing (LG), moderate grazing (MG), and heavy grazing (HG). Notes: * = SE could not be calculated due to limited degrees of freedom; NA = data unavailable for this species under the given treatment.

| **Host plant species** | **CK** | **LG** | **MG** | **HG** |
| --- | --- | --- | --- | --- |
| *Ajuga lupulina* | NA | NA | NA | 18.18* |
| *Aster altaicus* | 21.33 ± 4.07 | 8.89 ± 8.89 | 4.67 ± 4.67 | 24.00±2.31 |
| *Astragalus laxmannii* | 0.00 ± 0.00 | 0.00 ± 0.00 | 0.00 ± 0.00 | 6.67±6.67 |
| *Bupleurum chinense* | 96* | NA | NA | NA |
| *Carex aridula* | 92.00 ± 1.33 | 94.78 ± 0.89 | 86.22 ± 7.11 | 61.33±5.55 |
| *Dracocephalum heterophyllum* | 34.68 ± 2.06 | 20.00 ± 4.62 | 33.14 ± 8.05 | 19.83±6.13 |
| *Elymus nutans* | 52.00* | NA | NA | NA |
| *Kobresia humilis* | 90.22 ± 3.47 | 93.56 ± 5.67 | 78.67 ± 2.31 | 64.89±3.56 |
| *Koeleria cristata* | 75.11 ± 7.79 | 75.78 ± 2.89 | 61.93 ± 4.23 | 40.89±3.80 |
| *Leymus secalinus* | 70.45 ± 7.45 | 72.83 ± 1.65 | 36.39 ± 9.29 | 34.17±3.33 |
| *Medicago ruthenica* | 52.00* | 18.67 ± 9.33 | NA | NA |
| *Oxytropis subfalcata* | 2.00 ± 2.00 | 0.00 ± 0.00 | 0.00 ± 0.00 | 0.00±0.00 |
| *Poa annua* | 56.00 ± 12.02 | 61.78 ± 0.89 | 43.56 ± 5.24 | 45.78±3.47 |
| *Potentilla acaulis* | 64.00 ± 36.11 | 54.67 ± 4.07 | 41.11 ± 6.14 | 40.67±7.00 |
| *Potentilla bifurca* | 90.00* | 56.00 ± 18.3 | 46.08 ± 6.55 | 44.00±8.33 |
| *Potentilla multicaulis* | 70.22 ± 10.95 | 40.67 ± 9.26 | 36.89 ± 6.55 | 25.41±2.70 |
| *Sibbaldia adpressa* | 22.89 ± 3.45 | 70.00 ± 13.1 | 46.67 ± 6.3 | 69.47±3.27 |
| *Stellera chamaejasme* | 78.67 ± 1.76 | 64.89 ± 2.35 | 27.78 ± 8.00 | 57.33±11.65 |
| *Stipa tianschanica* | 59.56 ± 11.14 | 85.33 ± 1.54 | 44.44 ± 9.05 | 44.44±5.13 |
| *Thalictrum aquilegiifolium* | NA | 71.67 ± 8.33 | NA | NA |
| *Thermopsis lanceolata* | 20.00* | 32.00* | NA | NA |

**Table S3.** Mean ± standard error (SE) of the disease severity index for each species under control (CK), light grazing (LG), moderate grazing (MG), and heavy grazing (HG). Notes: * = SE could not be calculated due to limited degrees of freedom; NA = data unavailable for this species under the given treatment.

| **Host plant species** | **CK** | **LG** | **MG** | **HG** |
| --- | --- | --- | --- | --- |
| *Ajuga lupulina* | NA | NA | NA | 3.64* |
| *Aster altaicus* | 5.33 ± 0.96 | 3.11 ± 3.11 | 1.73 ± 1.73 | 5.87 ± 0.71 |
| *Astragalus laxmannii* | 0.00 ± 0.00 | 0.00 ± 0.00 | 0.00 ± 0.00 | 2.00 ± 2.00 |
| *Bupleurum chinense* | 37.60* | NA | NA | NA |
| *Carex aridula* | 38.93 ± 2.97 | 49.20 ± 1.07 | 29.24 ± 4.05 | 17.33 ± 2.12 |
| *Dracocephalum heterophyllum* | 11.89 ± 1.46 | 7.20 ± 2.01 | 8.01 ± 2.26 | 4.59 ± 1.72 |
| *Elymus nutans* | 18.40* | NA | NA | NA |
| *Kobresia humilis* | 42.13 ± 2.13 | 48.02 ± 1.43 | 28.71 ± 2.45 | 16.98 ± 1.11 |
| *Koeleria cristata* | 27.16 ± 3.86 | 26.58 ± 0.49 | 21.04 ± 3.93 | 11.56 ± 0.69 |
| *Leymus secalinus* | 23.82 ± 6.67 | 27.12 ± 1.67 | 11.94 ± 5.71 | 9.67 ± 1.02 |
| *Medicago ruthenica* | 22.40* | 4.53 ± 2.28 | NA | NA |
| *Oxytropis subfalcata* | 0.40 ± 0.40 | 0.00 ± 0.00 | 0.00 ± 0.00 | 0.00 ± 0.00 |
| *Poa annua* | 17.51 ± 5.13 | 19.29 ± 1.25 | 13.24 ± 1.72 | 15.53 ± 0.64 |
| *Potentilla acaulis* | 27.73 ± 4.13 | 17.33 ± 1.89 | 17.11 ± 1.56 | 13.60 ± 2.74 |
| *Potentilla bifurca* | 37.20* | 19.87 ± 8.03 | 12.58 ± 4.29 | 13.60 ± 5.25 |
| *Potentilla multicaulis* | 25.56 ± 3.91 | 16.27 ± 2.15 | 10.00 ± 2.43 | 7.70 ± 1.14 |
| *Sibbaldia adpressa* | 7.24 ± 0.60 | 24.27 ± 3.98 | 11.91 ± 2.39 | 20.17 ± 1.98 |
| *Stellera chamaejasme* | 36.27 ± 2.54 | 18.49 ± 0.58 | 7.64 ± 2.62 | 16.18 ± 3.97 |
| *Stipa tianschanica* | 20.53 ± 4.27 | 28.80 ± 0.56 | 13.16 ± 3.72 | 13.07 ± 1.75 |
| *Thalictrum aquilegiifolium* | NA | 21.07 ± 1.87 | NA | NA |
| *Thermopsis lanceolata* | 4.00* | 10.04* | NA | NA |

**Table S4.** Results of linear mixed-effects models examining the effects of grazing intensity (light, LG; moderate, MG; and heavy, HG) on community foliar disease incidence (%), community foliar disease severity, total pathogen relative abundance, pathogen richness, *Alternaria* absolute abundance (gene copies g^-1^), and the relative abundance (%) of *Alternaria*, *Fusarium*, and *Paraphoma*. Significant effects (*P* < 0.05) are shown in bold.

| **Response** | **LG** | | | **MG** | | | **HG** | | |
| --- | --- | --- | --- | --- | --- | --- | --- | --- | --- |
|  | Estimate | *t* | *P* | Estimate | *t* | *P* | Estimate | *t* | *P* |
| Community foliar disease incidence (%) | **11.86** | **3.58** | **0.001** | **-11.03** | **-3.33** | **0.002** | **-23.30** | **-7.03** | **< 0.001** |
| Community foliar disease severity | **5.89** | **3.08** | **0.004** | **-7.69** | **-4.03** | **< 0.001** | **-12.73** | **-6.67** | **< 0.001** |
| All pathogens (%) | **8.76** | **7.20** | **< 0.001** | -2.13 | -1.75 | 0.090 | 2.72 | 2.23 | 0.053 |
| Pathogen richness | 3.67 | 0.74 | 0.465 | 5.00 | 1.01 | 0.320 | -2.89 | -0.58 | 0.564 |
| *Alternaria* (gene copies g^-1^) | **0.77** | **7.06** | **< 0.001** | 0.09 | 0.79 | 0.440 | 0.11 | 1.01 | 0.323 |
| *Alternaria* (%) | **0.32** | **7.85** | **< 0.001** | -0.03 | -0.62 | 0.540 | 0.04 | 0.98 | 0.337 |
| *Fusarium* (%) | **2.25** | **7.43** | **< 0.001** | **-0.70** | **-2.30** | **0.028** | 0.11 | 0.36 | 0.724 |
| *Paraphoma* (%) | -0.12 | -0.31 | 0.758 | **-0.96** | **-2.48** | **0.020** | **1.57** | **4.05** | **< 0.001** |

**Table S5.** Results of linear mixed-effects models examining the effects of grazing intensity (light, LG; moderate, MG; and heavy, HG) on soil properties. AP, available phosphorus; NH_4_^+^-N, ammonium nitrogen; NO_3_^-^-N, nitrate nitrogen; SOC, soil organic carbon; TN, total nitrogen; TP, total phosphorus. Significant effects (*P* < 0.05) are shown in bold.

| **Response** | **LG** | | | **MG** | | | **HG** | | |
| --- | --- | --- | --- | --- | --- | --- | --- | --- | --- |
|  | Estimate | *t* | *P* | Estimate | *t* | *P* | Estimate | *t* | *P* |
| AP | 0.92 | 1.25 | 0.225 | 0.94 | 1.27 | 0.22 | 1.49 | 2.01 | 0.06 |
| NH_4_^+^-N | **2.29** | **2.88** | **0.008** | **2.02** | **2.55** | **0.02** | **4.98** | **6.26** | **< 0.001** |
| NO_3_^-^-N | -1.52 | -2.06 | 0.051 | **-2.62** | **-3.56** | **0.00** | **-3.38** | **-4.60** | **< 0.001** |
| SOC | **10.93** | **2.66** | **0.012** | **10.55** | **2.16** | **0.04** | 4.93 | 1.20 | 0.24 |
| TN | **0.33** | **3.27** | **0.003** | **0.41** | **4.08** | **< 0.001** | **0.23** | **2.26** | **0.03** |
| TP | 0.02 | 0.96 | 0.344 | 0.04 | 1.68 | 0.10 | 0.03 | 1.01 | 0.32 |

**Figure S1.** Leaf spot symptoms and colony morphology of two dominant species, *Kobresia humilis* (a–c) and *Carex aridula* (d–f). (a, d) field symptoms; (b, e) colony morphology on PDA after 7 days at 23 °C (obverse); (c, f) reverse view. Both pathogens were identified as *Alternaria* spp.

**Figure S2.** Relative abundance of the most common soil fungal pathogens (proportion of total ITS sequences).
